# Risk factors of caregiver burden among caregivers of older people in rural eastern Uganda

**DOI:** 10.1002/alz70860_103928

**Published:** 2025-12-23

**Authors:** Stephen Ojiambo Wandera, Monica M Diaz, Shafiq Kawooya, Lodrick Wabwire Odo, Noeline Nakasujja

**Affiliations:** ^1^ Makerere University, Kampala, Central, Uganda; ^2^ University of North Carolina at Chapel Hill School of Medicine, Chapel Hill, NC, USA; ^3^ Makerere University, Kampala, Uganda

## Abstract

**Background:**

The population of adults aged 60 years and older in sub‐Saharan Africa (SSA) is projected to reach 670 million by 2030. In Uganda, this age group makes up about 5% of the population. The increasing number of older persons presents unique social and healthcare challenges, including non‐communicable diseases (NCDs) like frailty, multimorbidity, disability, and dementia, which are under‐studied in SSA, especially among community‐dwelling older persons in rural Uganda. This study aimed to determine the prevalence and factors associated with caregiver burden among caregivers of older persons with and without dementia in rural eastern Uganda

**Method:**

A cross‐sectional study was conducted in rural eastern Uganda (Busia and Namayingo districts) from December 2023 to September 2024. Cognitive impairment in older persons was assessed using the IDEA and Rowland University Dementia Assessment Scale (RUDAS). Caregiver burden was measured with the Zarit Caregiver Burden Interview scale, and caregiver depression with the Patient Health Questionnaire‐9. Frequencies and multivariable logistic regression analyses were performed.

**Result:**

The study included 602 dyads of older persons and their caregivers (mean age 38.1 years; 65% female; mean education level 1.2 years). Most caregivers were adult children (40.8%), spouses (25%), or adult grandchildren (17%). Caregiver burden was prevalent in 62% of caregivers (44% moderate, 18% severe; mean score 12.9). The mean PHQ‐9 score was 9.8. Multivariable regression showed that older caregiver age, being a son, less time knowing the older person, and higher PHQ‐9 scores were associated with greater caregiver burden.

**Conclusion:**

There is a high prevalence of caregiver burden in rural Uganda, with caregiver depression and older age being significant factors, while income and education were not risk factors. Caregiver education and targeted risk reduction could help alleviate caregiver burden and reduce dementia in rural SSA.

